# Making It Work: Physicians' Perspectives on the Rapid Transition to Telemedicine

**DOI:** 10.1089/tmr.2020.0038

**Published:** 2021-05-03

**Authors:** Matthew J. DePuccio, Alice A. Gaughan, Ann Scheck McAlearney

**Affiliations:** ^1^ CATALYST, Center for the Advancement of Team Science, Analytics, and Systems Thinking, College of Medicine, The Ohio State University, Columbus, Ohio, USA.; ^2^ Department of Family and Community Medicine, College of Medicine, The Ohio State University, Columbus, Ohio, USA.; ^3^ Department of Biomedical Informatics, College of Medicine, The Ohio State University, Columbus, Ohio, USA.

**Keywords:** implementation science, primary care, qualitative research, telemedicine

## Abstract

**Background:** Telemedicine is a major pillar in the health care system's response to the coronavirus disease 2019 (COVID-19) pandemic. However, the rapid implementation of telemedicine is not without its challenges. We examined the strategies primary care physicians (PCPs) used to make the transition to telemedicine during the pandemic.

**Methods:** A qualitative study was conducted to explore the perspectives of PCPs working at a Midwestern Academic Medical Center (AMC) who used telemedicine during the COVID-19 pandemic. Semistructured interviews with 20 PCPs were conducted 3 months following the rapid increase in the use of telemedicine across the AMC. Interview questions asked about physicians' challenges using telemedicine, the changes they had to make to use telemedicine, and what had helped them deliver care through telemedicine. All interviews were recorded, transcribed, coded, and rigorously analyzed using deductive thematic analysis.

**Results:** According to PCPs, a successful transition to telemedicine involved three key elements: (1) maintaining flexibility in the context of constant change; (2) recognizing the need to upgrade their home office spaces; and (3) seeking opportunities to continue collaborating and sharing knowledge with peers. These strategies enabled physicians to rapidly pivot to deliver care through telemedicine when stay-at-home orders took effect. Physicians also described how frequent leadership communication and the rapid dissemination of telemedicine training supported their use of this care modality.

**Conclusions:** Successful adoption of telemedicine requires that physicians adapt their care delivery practices. Considering these facilitators of telemedicine use can help both physicians and health care organizations with this important transition.

## Introduction

In response to the coronavirus disease 2019 (COVID-19) pandemic, primary care practices across the U.S. rapidly transitioned from in-person patient visits to telemedicine.^1,2^ According to the National Academy of Medicine, telemedicine is “the use of electronic information and communications technologies to provide and support health care when distance separates the participants.”^3^ Through the use of technologies such as personal smart devices, computers, and telephones, telemedicine has been instrumental in minimizing the spread of the novel coronavirus while also supporting the delivery of necessary health services. Particularly, physicians can leverage patient portals and electronic medical record (EMR) systems to help patients monitor their health, share updated information about their care plans, and access educational materials to manage chronic diseases.^4^ As a result, telemedicine has the potential to improve health outcomes and reduce unnecessary health care utilization^5,6^ by making it possible for patients to become active participants in their health care.^7^ Moreover, the expansion of telemedicine reimbursement more broadly during the COVID-19 pandemic^8^ also suggests policymakers and payers recognize that telemedicine is an important care delivery approach that can limit the community spread of the novel coronavirus while enabling the delivery of safe and convenient care to patients and families.

Despite the potential advantages of telemedicine, primary care physicians' (PCPs) rapid transition to care delivery through these modalities in response to the emergence of COVID-19 has proven difficult. In one early study, PCPs reported having to frequently adjust their workflows, reexamine staffing models, and develop solutions to ensure patients were prepared for their virtual visit.^9^ There have also been multiple reports of physicians and staff having to provide patients with technical assistance or needing to convert video visits to telephone visits in cases where patients have not been able to log into a telemedicine visit.^10,11^ As the delivery of primary care through telemedicine has continued, it is important to understand what has helped PCPs and their practices overcome the challenges of this rapid transition.

While previous research has examined the initial implementation and expansion of telemedicine modalities as a result of the emergence of COVID-19,^9,12–14^ we have limited understanding of how physicians have adapted to facilitate their use of telemedicine as the pandemic has continued. This qualitative study aims to improve our understanding about the early adjustments made by PCPs to successfully deliver care through telemedicine during the pandemic. As implementation is typically considered an ongoing process,^15^ insight about these adaptations, which may include new or modified workflows and safety protocols, can be applied to help PCPs and their practices in their efforts to use telemedicine both during the COVID-19 pandemic and into the future. Our study focused on the perspectives of PCPs because these providers have first-hand experience using and adapting their use of telemedicine, which can inform future implementation efforts. Furthermore, as these changes in practice and workflows will likely have an impact on the delivery of primary care,^16^ both physicians and organizations can benefit from learning about specific practices that can potentially facilitate the use of telemedicine and ensure the delivery of high-quality care through telemedicine modalities. Therefore, the specific research question we set out to answer was, how did physicians describe overcoming the obstacles to using telemedicine during the COVID-19 pandemic?

## Methods

### Study setting and sample

We initiated a qualitative study as primary care practices associated with a large Midwestern Academic Medical Center (AMC) shifted to providing care through telemedicine following the emergence of COVID-19. In response to the pandemic, in March 2020, the AMC convened a telemedicine work group to oversee the expansion of telemedicine and to develop recommendations for using telemedicine in this context. While the workgroup attempted to remove barriers to the widespread adoption of telemedicine, physicians were encouraged to use the variety of electronic means available (e.g., patient portal, telephone, and video) to deliver care through telemedicine. Initially, in the early phases of the pandemic, most primary care visits occurred through telemedicine. Of these telemedicine appointments, most were completed through Epic MyChart or Doximity, but additional platforms such as Zoom and FaceTime were also made available to accommodate the technological needs of patients and physicians.

With the approval of clinical leaders, we sent email recruitment letters to 106 PCPs working in General Internal Medicine (GIM) or Family and Community Medicine (FCM) practices within the AMC in June 2020. The recruitment emails, which explained the purpose of our voluntary study and the criteria for participating, were sent to PCPs, and those interested in participating replied to our email and indicated their availability for an interview. Participants did not receive compensation for participating in this study. The Institutional Review Board of the Ohio State University approved this study.

### Data collection

Two coauthors M.J.D. and A.S.M. conducted one-on-one video interviews with 20 PCPs between July and August 2020. We used a semistructured interview guide that asked questions about the early impacts of COVID-19 on their work as PCPs and what they perceived to be major challenges with delivering primary care through telemedicine during the pandemic. We also asked how the changes brought about by COVID-19 affected the way physicians worked with their usual staff and care teams. A copy of the interview guide is available upon request from the corresponding author.

Video interviews lasted ∼25 min on average and all interviewees provided informed consent to participate in the study. All interviews were audio recorded, transcribed verbatim, and deidentified. Interviewees' years of experience in primary care ranged between 3 and 41 years with an average of 16 years. The majority of interviewees were female (55%) and worked in the FCM department (65%).

### Data analysis

Interview transcripts were analyzed using a deductive dominant thematic analysis,^17,18^ allowing for categorization of data based on general themes derived from the interview guides, as well as identification of emergent themes; this approach ensured that we reached saturation in data collection. Our approach also allowed for comparison of themes across interviews and enabled us to characterize the ways PCPs facilitated their transition to delivering care through telemedicine as we present in this study.

We used ATLAS.ti (version 8.4.4) qualitative data analysis software to support our coding and analysis process. More specifically, two coauthors M.J.D. and A.A.G. reviewed all of the transcripts independently and developed an initial codebook based on questions asked in the semistructured interview guide and other topics that emerged during the initial review of the transcripts. The refined and final codebook captured topics centered around the impact of COVID-19 on primary care delivery as well as the changes that were necessary to facilitate telemedicine use during the pandemic. These two coauthors M.J.D. and A.A.G., supervised by A.S.M., agreed on the definitions included in the codebook and A.A.G. independently coded each interview. All three authors met weekly to discuss the emergent findings and came to consensus on the final themes we describe below.

## Results

The PCPs we interviewed described both individual adjustments they had to make and how their organizations supported their efforts to “make telemedicine work” with their rapid transition to delivering virtual care with the emergence of COVID-19. In the following sections, we describe the adjustments PCPs reported necessary to transition to telemedicine modalities, as well as how their organization and its leaders supported the rapid expansion of telemedicine use across the institution.

### Individual adjustments to facilitate use of telemedicine

When discussing their rapid transition to using telemedicine in the context of the pandemic, PCPs described three key elements of this transition: (1) maintaining flexibility in the context of constant change; (2) recognizing the need to upgrade their home office spaces; and (3) seeking opportunities to continue collaborating and sharing knowledge with peers. Below we elaborate on these three types of adjustments, and present additional example quotations in Table 1. We use a letter at the end of each quotation to protect the identity of each interviewee.

**Table 1.** Individual Adjustments Facilitating Rapid Expansion of Telemedicine Use During the Coronavirus Disease 2019 Pandemic

Theme	Perspectives of PCPs
Flexibility in the face of change	I think the change in the way that we deliver care is good for the most part. I mean, I think everybody's learned that there's a lot of different ways that we can deliver care. (E)
	Sometimes I can say ‘hey, let me follow up with you in a few days’ instead of having them come into the office. I can call them up and do a virtual visit again. I have a little bit more flexibility… I don't have to have clinic hours where I'm kind of restricted, I do… I mean I have those two half days blocked off. But if … if we can't, if something happens, we can't connect, I can have the staff reschedule the appointment the next day because I'm doing other things, it's easy for me to get online and reach out to them that way. (R)
Upgrading work spaces at home	Well, my desktop didn't have a computer or a camera before and here I am with my desktop with a camera. That's a new resource that I am using right now while I talk to you. (K)
	I bought this little light thing to enhance it, but it's still not the best lighting component, so we had to with our Zoom meetings and we had to do a lot of faculty development ourselves of how to set up the setting and working on that component. And I think it's important from our perspective that when we are talking to patients that you know, they can see us well and have that piece. I mean you are lit up pretty well. (R)
Peer-to-peer collaboration and knowledge sharing	I think from a divisional perspective, everybody kind of pulling together and coming up with smart phrases and things like that that the entire faculty group could use so that nobody was kind of going re-inventing the way they were doing things. (E) We have a messaging thread with all of the providers. So I'll pull up the thread and shoot a message and typically I get a response within a half an hour to an hour or so. (F)
	We are a pretty tight-knit group and there are like 50 or 60 of us now but like I can reach out to anyone in the division and they'll walk us through it, which is really nice. So once a couple of us were trained in our office, we just taught everyone how to do it. (T)

Letters in parentheses denote the specific interviewee.

PCP, primary care physician.

#### Maintaining flexibility in the face of change

In the wake of the pandemic, physicians indicated that the sudden shift to telemedicine necessitated they “figure out” and learn new ways of delivering primary care. This flexibility involved both adapting the way they delivered care to patients by learning to use new technologies, and being more flexible in the ways they approached and attempted to address patient needs and concerns. As one physician explained, “I'm still delivering the same care for the same problems, but just doing so in a different manner that has necessitated figuring out how to do certain things that perhaps we did not think we could do. So, the overall care itself, I would say is the same, the manner in which we provide it is what differs.” (B) Another physician explained, “When it comes to virtual visits, making allowances and being a little bit more flexible about when you can call a patient, when you can talk with them. I think it has been helpful for the patient as well as for myself as far as when I'm available and able to make the phone calls, so I think having some flexibility has definitely helped me.” (L)

Some physicians mentioned that their comfort and familiarity with a variety of telemedicine platforms and technological devices made it easier to perform tasks such as scheduling appointments, sending and receiving patient messages to facilitate virtual visits, and even participating in department meetings. One physician explained, “I'm fairly comfortable using technology, which has helped me a great deal. I mean, I learned, within the first day of telemedicine, how to make appointments. If you're not comfortable playing with [the Integrated Health Information System], there's a lot of functionalities that you're not using that can make your life so much easier.” (I) Another physician shared, “I didn't feel like I even had a transitional period. I was already living my life with a smart device as the smart iPhone, the smart iPad that I was already Zooming with a lot of people, and Webexing because being on campus meetings.” (O)

#### Upgrading work spaces at home

Making home work environments conducive for telemedicine visits was also noted by many physicians as an important change that was necessary to implement telemedicine. One physician noted, “I think we've all spent time upgrading our home work environments.” (G) Another physician similarly shared, “There's a particular room in my house that I have really good light and really good, you know, really good visibility. So, it's a place that I like to do the visits from, but like it took me a while to figure out where the right place was to do that. I had to test it out a few different times, and what was the most professional and won't have as many distractions.” (M) Physicians also indicated that these modifications allowed them to pivot to delivering telemedicine visits in the early months of the pandemic when stay-at-home orders were in effect to protect patients, physicians, and other health care providers from the community spread of the coronavirus.

#### Peer-to-peer collaboration and knowledge sharing

Although some PCPs were already familiar with and were using telemedicine in their practice before the pandemic, several interviewees explained how they relied on colleagues, and sometimes even their students, for advice on how to use the different technologies that became available during the telemedicine transition. A physician explained, “Okay, actually the pharmacist who works in our office. They've been doing televideo visits for over a year and a half so they are really good. And so, she's been a huge resource for me to talk about, you know, how do we do this? What are some things that work and stuff like that? So yeah, so talking with your, you know, your colleagues and trying to get tips from them is good.” (A) Another physician shared, “I learned so much from the students. And that's another factor that helped me … the students taught me about the Slack app… and the Doodle app and all of the everything has an app, right? So, I felt like working with the youngsters helps a lot really.” (O)

Technological tools, such as secure instant messaging applications, were viewed as advantageous because they fostered collaboration and knowledge sharing between physicians and other staff members. One physician explained, “So all day long, we have these messages that just pop up and what would you do? Let me run this by you. And yeah, so I think that's another resource that is very useful.” (I) In turn, PCPs were able to streamline communication with other team members while working remotely, which allowed them to respond to emergent patient questions or workflow issues. For instance, a PCP provided an example of how they used secure messaging to relay a patient concern: “We started using…our secure message[s]… We started using that a lot more to sort of communicate in a you know minute by minute…. They'd be like, ‘Miss Jones is calling in saying she hasn't heard from you about her appointment. It's 10 minutes after appointment.’ I'm like, ‘Ten minutes.’ She's really freaking out about that.” (N) Physicians also described how providers developed electronic repositories that contained different “smart phrases” (i.e., standardized text that can be copied and pasted into the EMR) that other providers could access and that could improve the consistency and efficiency of documentation (Table 1).

### Organizational support for transitioning to telemedicine

Given their need to rapidly transition to the delivery of telemedicine, physicians also reflected upon how support from their organizations helped them with their adjustment. In particular, physicians described how (1) frequent leadership communication and (2) the rapid dissemination of telemedicine training were important factors that supported their transition to using telemedicine. Below we describe each of these types of organizational support in more detail, and in Table 2 we present additional supporting quotations from the interviewees.

**Table 2.** Organizational Factors Supporting Telemedicine Use During the Coronavirus Disease 2019 Pandemic

Theme	Perspectives of PCPs
Frequent leadership communication	That [daily pep talk from leadership] was so informative and really helpful to just kind of see where we were as an organization, what's opening now, what procedures can we do and all those kind of things. (A)
	I would say that our lead physician at X was amazing. Doctor X was sending weekly updates, making sure that the plans are well communicated, worked very well with the clinic manager to create the secure chats group. So I feel like having a leadership that recognizing that this is real and it's happening and it's probably here to stay and it's not going anywhere and being able to pick up your balance in a timely fashion and really show leadership skills have been a great opportunity and huge tool to adapt to the changes. (O)
Rapid dissemination of telemedicine training	We had a good bit of training at the beginning, a couple of our faculty who are super users in Epic provided some videos of how to access video visits, we had billing training around you're using these different codes for telemedicine. We had different training around ways we came to telemedicine and what type of component and how to access those platforms and training… I think that there's been actually in our system an extensive amount of training… I feel very comfortable with the tools and I feel comfortable with the billing piece of it because we've had an ongoing guidance. (K)
	We create a lot of like tip sheets and workflow things for people to train on their own, because that's the other with the pandemic, you couldn't necessarily bring people in a room and have them train or have trainers go to the practices and meet with people. Like you have to do a lot of self-directed learning and training for people. (M)

Letters in parentheses denote the specific interviewee.

PCP, primary care physician.

#### Frequent leadership communication

Regular communication from leadership was important because it was a means through which physicians could stay up to date with rapidly changing institutional guidelines about telemedicine. One physician explained, “[Leadership] was very good about a daily email about, you know, kind of what was going on, and very reassuring about what things were going into play, and they were very good about communicating what changes were happening.” (E) These messages reportedly helped PCPs feel supported in their efforts to implement telemedicine as they also provided timely information about how they could optimize telemedicine in the face of rapidly changing COVID-19 policies and protocols. Another PCP noted:

I think there was a lot of support. I think having institutional support and departmental support for telemedicine and some honest recognition that you know, things were crazy and that we needed to move to more rapid, you know have more rapid changes take place and getting a lot of that reassurance. I thought that helped a lot. There were constantly emails coming out about how to best optimize telemedicine and you could tell that groups are working on efficient products and you know it might not have been perfect but people were really trying to make it as easy as possible. So, I thought that was really helpful. (H)

#### Rapid dissemination of telemedicine training

New training provided by the AMC, including recommendations from the organization's FCM and GIM telemedicine working groups, was also noted by many physicians as instrumental for supporting their expanded use of telemedicine. Trainings prioritized topics such as how physicians should use third-party applications to protect patients' privacy and how to document services in the EMR so that the physicians would be reimbursed for telemedicine encounters. As one interviewee reflected:

Our department did a fantastic job of pushing out trainings. [They] were reviewing, synthesizing, references, and resources…I think there was a real effort to do it and the reality is like there was enough downtime in the beginning when [patients] just weren't showing up… The multiple modalities of the third-party video software versus the internal software and training on what needs to be done, like what needs to be documented…, and adoption of those things. (H)

Another physician further explained, “They made sure that everybody knew how to log on because you have to log on differently when we went to third party apps… And then those in the department who were responsible made sure that we were getting education on how to utilize Updox, utilize Doximity, and here's how to utilize those, including when we had students come back.” (B)

## Discussion

The COVID-19 pandemic forced a rapid increase in the use of telemedicine in primary care as well as in other health care settings.^19,20^ Our qualitative study suggests that maintaining flexibility, modifying work spaces, and taking advantage of opportunities to collaborate virtually helped PCPs adjust to delivering care virtually through telemedicine during the early phases of the COVID-19 pandemic. Similarly, organizational support through means, such as frequent communications from leadership and providing training, were also key in facilitating this telemedicine transition in response to the emergence of COVID-19. As the use of telemedicine is likely to continue, our findings may help support PCPs and their practices in their ongoing efforts to implement and use telemedicine.

In light of the practice and workflow changes brought about by the COVID-19 pandemic,^21,22^ we found that PCPs were able to remain flexible in terms of how they delivered telemedicine to better accommodate patients' needs and preferences. Our data also revealed that PCPs' familiarity with technology enabled them to better respond to patients' needs in the context of rapid telemedicine implementation. Insight about the ways physicians adjusted during the expansion of telemedicine use, such as how they rapidly learned about and navigated the variety of telemedicine platforms (e.g., video, telephone, or patient portal), may help inform future efforts to support telemedicine implementation and use. Although ongoing telemedicine trainings may potentially reinforce these types of adjustments,^23^ understanding the changes physicians have had to make to accommodate the increased use of telemedicine can also offer invaluable information about ways to improve the efficiency and effectiveness of care delivered through telemedicine. For instance, given our interviewees' enthusiasm about the use of messaging tools to rapidly share information with other physicians, our findings suggest it will be important to emphasize the opportunity for bidirectional communication to facilitate problem solving and knowledge sharing when using telemedicine.^24^ Furthermore, as the demand for telemedicine during the COVID-19 pandemic increased,^19,25^ future inquiries may want to examine the long-term benefits of “how-to” knowledge sharing that occurred as the pandemic progressed and ultimately wanes. It may be that secure messaging platforms enable physicians and other health care providers to share their technological as well as their clinical expertise in a way that fosters the dissemination of best practices that can make the ongoing use of telemedicine easier for physicians and their patients.

Our research may also have important managerial implications. Frequent leadership communication and rapid dissemination of telemedicine training, as described in our study, represent two ways managers can facilitate telemedicine use. Given the prioritization of telemedicine during the pandemic, managers play an important supporting role ensuring physicians have an up-to-date understanding of the different organizational policies and procedures related to telemedicine use.^26,27^ As was the case in other health care organizations,^9^ physicians in our study acknowledged the importance of executive and clinical leadership in disseminating information about available trainings and encouraging the optimal use of telemedicine. Thus, our findings demonstrate that AMC leadership can facilitate physicians' use of telemedicine by disseminating information from technical experts and conveying the importance of properly using telemedicine platforms (e.g., Doximity). In fact, based on previous reports,^28^ health care organizations may want to consider making technical experts available to assist physicians with workflow issues and thus mitigate technological barriers to telemedicine use in the future.

Given the sudden shift to telemedicine modalities driven by the emergence of COVID-19, our findings are likely to resonate with PCPs and other types of health care providers who had to adapt how they delivered patient care during the pandemic. Looking ahead, as physicians are now shifting from virtual-only to more hybrid models of patient care where both in-person visits and telemedicine are used, it will be critical for researchers and practitioners to consider how the adjustments physicians made that were identified in this study will continue to affect physicians' abilities to use telemedicine, as well as whether new adjustments are needed. It will also be important for organizational leaders to take into account the strategies we describe in this article that can help support physicians in their ongoing use of telemedicine. Organizations may also want to consider building flexibility into ongoing telemedicine support plans.^29^ As our study has shown, PCPs have had to adapt their practice approaches as well as their surroundings to make telemedicine work, and these findings should be considered as organizations continue to assess what adjustments are needed to support the ongoing use of telemedicine.

### Limitations

Our study has several limitations that are worth noting. First, given the qualitative nature of our study, we are unable to evaluate the extent to which the approaches we identified actually impacted telemedicine use in primary care. Future research can be conducted to examine the association between these approaches and telemedicine use metrics such as the appropriateness and frequency of use of telemedicine for different patient groups or conditions. Second, as this study was focused on the primary care practices associated with a single AMC, it is possible that the perspectives of providers in other settings may vary. Despite our recruitment of a relatively small number of providers from a single AMC, our data collection strategy enabled us to reach saturation with respect to the themes we report. We are confident that the findings we present are salient and hope these results can provide insight for other PCPs and organizations attempting to facilitate expanded use of telemedicine modalities. Third, we acknowledge that the interpretation of our findings may be influenced by self-selection bias as study participants may have been more technologically savvy and/or more willing to share their experiences with telemedicine implementation than those who did not choose to participate. Finally, given the variability in levels of clinical experience across our interviewees, future study is warranted to explicitly examine how the adaptations made by PCPs might be differently described by those with less clinical experience compared with those with more clinical experience.

## Conclusions

Our study shows that physicians' adjustments to facilitate their use of telemedicine have helped them to deliver care virtually during the COVID-19 pandemic. Maintaining flexibility, upgrading home office spaces, and collaborating virtually with colleagues appear to be important elements of this rapid transition from in-person care to telemedicine. At the same time, from an organizational standpoint, leaders who can share timely information and provide training on how to best use telemedicine may help minimize barriers to telemedicine use. As primary care practices continue to use telemedicine both during the COVID-19 pandemic and beyond, continuing research is needed to understand how to sustain the long-term use of telemedicine and how to support PCPs as they work to address the needs of their patients.

## Abbreviations Used

AMC: Academic Medical Center
COVID-19: coronavirus disease 2019
EMR: electronic medical record
FCM: Family and Community Medicine
GIM: General Internal Medicine
PCP: primary care physician
